# Reply to: Addressing racial and ethnic disparities in AACR project GENIE

**DOI:** 10.1038/s41698-023-00426-4

**Published:** 2023-08-31

**Authors:** Alexander T. M. Cheung, Andrzej Niemierko, Eliezer Van Allen, Neha Vapiwala, Sophia C. Kamran

**Affiliations:** 1 NYU Grossman School of Medicine, New York, NY USA; 2 Broad Institute of Harvard and MIT, Cambridge, MA USA; 3 Department of Radiation Oncology, Massachusetts General Hospital, Harvard Medical School, Boston, MA USA; 4 Dana-Farber Cancer Institute, Boston, MA USA; 5 Department of Radiation Oncology, Hospital of the University of Pennsylvania, Perelman School of Medicine, Philadelphia, PA USA

**Keywords:** Cancer genomics, Biomarkers

**replying to** Sweeney et al. *npj Precision Oncology* 10.1038/s41698-023-00425-5 (2023)

We thank Sweeney et al. for their comments regarding our recent article evaluating the representation of racial/ethnic minority groups in the AACR Project GENIE for precision oncology research. Our findings^1^ suggest that this real-world biorepository—while providing a wealth of clinical-genomic data—may not accurately reflect the actual distribution of various cancer types in the general United States patient population.

Sweeney et al. disagree with our “emphasis on powering comparisons for a Cohen’s h of less than 0.2.” However, these benchmark values of 0.2, 0.5, and 0.8 for “small,” “medium,” and “large” effect sizes, respectively, are arbitrary and should not be interpreted rigidly^2^, with different thresholds depending on the scientific study^3^; Cohen in fact warns against inflexibility with respect to these values^4,5^. Sawilowsky expanded the benchmarks and definitions to range from 0.01 (very small) to 2.0 (huge), based on updated research findings in the applied literature^4^. We explicitly defined a small effect size as a Cohen’s h less than or equal to 0.2 and used this definition to identify, in comparison to white samples, which racial/ethnic groups within the GENIE database have insufficient sample sizes for studying small genomic differences. Moreover, since genomic differences between cancer phenotypes often occur on a long-tail distribution^6^, we anticipated that researchers might be interested in even smaller and subtler effect sizes. Regardless of the 0.2 vs. <0.2 cutoff, there would be no change expected in the overarching conclusions, which are (i) that the dataset is underpowered for comparisons of Black, Asian, and Hispanic primary and metastatic tumor samples versus white samples in two of five common cancers (prostate and pancreatic), and (ii) the dataset is also underpowered for comparisons of Native American and Pacific Islander samples versus white samples for all five common cancers that we evaluated in the primary and metastatic setting. Furthermore, for less common cancers, the lack of adequate representation within AACR Genie is more striking, as demonstrated in a recent analysis of pancreatic neuroendocrine tumors^7^.

We agree that it’s important to note that GENIE is a dynamic database and that analyses in our study were based on the version cited (v9.1-public release, January 2021). We were pleased to note that the latest release of GENIE (v13.0-public) contains more diverse and representative patient samples. However, our findings remain relevant and novel for the literature for several reasons. First, our study provides a snapshot of the GENIE database at a certain point in time, and our expectation is that future researchers can likewise audit the data and assess its evolution over successive iterations. Second, while the repository changes every six months, it is unlikely that all groups utilizing GENIE will be able to download, process, and analyze the data—not to mention the time required for peer review and actual publication—within that same timeframe. Indeed, we felt it was important to perform this analysis given several publications that used earlier versions of the GENIE database to draw conclusions about race and cancer genomics in poorly represented populations^8–10^ (occasionally with conflicting results)^11,12^, while also demonstrating its strengths in other patient subgroups and cancers for researchers interested in exploring questions in those contexts. We hope our findings provide a frame of reference for readers interpreting and evaluating published studies that employed this version and also encourage continued contributions from centers to help strengthen the registry in areas of need.

Lastly, we agree with Sweeney et al.’s point that biorepositories “*may not represent the broader population of patients that are diagnosed and treated for cancer*” due to barriers like institutional and participation biases. This also pertains to the GENIE registry, which currently reflects patient populations and practice patterns only at participating institutions. In fact, this was the impetus for our manuscript. We commend AACR for their dedicated efforts to improve this representation through a variety of initiatives and look forward to seeing this already valuable public biorepository grow into a more diverse and representative database for future generations of scientists, clinicians, and, most of all, patients.

## Data Availability

The data underlying the original article^1^ are available via the publicly available Project GENIE at genie.cbioportal.com, with additional information regarding its initial release found here: 10.1158/2159-8290.CD-17-0151.
